# Enhanced Genetically Variant Peptide Profiling from Human Hair Using Multiple Enzymes

**DOI:** 10.1021/acsomega.6c04197

**Published:** 2026-06-29

**Authors:** Zheng Zhang, William E. Wallace, Guanghui Wang, Meghan C. Burke, Stephen E. Stein

**Affiliations:** Mass Spectrometry Data Center, Biomolecular Measurement Division, 10833 National Institute of Standards and Technology, 100 Bureau Drive, Gaithersburg, Maryland 20899, United States

## Abstract

Genetically variant peptides (GVPs) derived from human hair proteins have been studied as evidence for forensic human identification. For this purpose, trypsin is generally routinely used as the protease for peptide generation. We hypothesized that the use of different proteases can expand sequence coverage in the hair proteome and, therefore, increase the chance of a variant peptide being identified and confirm the findings from tryptic digests. Since keratins and keratin-associated proteins are the main components of the hair proteome, we examined several alternative enzymes other than trypsin, including keratinase, chymotrypsin, and Glu-C. We found that the use of multiple enzymes led to enhanced GVP profiling and, therefore, an increase in the confidence of GVP identification.

## Graphical abstract

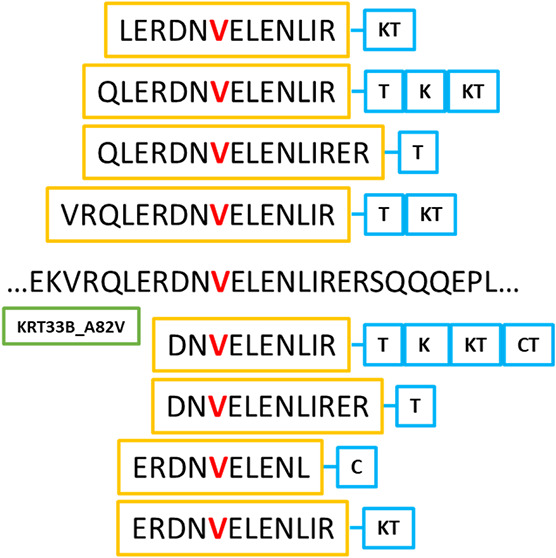

## Introduction

Genetically variant peptides (GVPs) derived from human hair proteins have been proposed and studied for use as evidence for human identification in forensics.^1,2^ A significant impediment to this goal is the difficulty in digesting hair due to the high degree of cross-linking and poor solubility of hair keratins (KRTs) and keratin-associated proteins (KRTAPs).^3,4^ In prior work, we developed a method called the direct extraction (DE) method^5^ to efficiently extract hair proteins from a single 5 cm-long hair strand. This method simplified protein extraction by requiring only a single step and taking 30 min.

At the time that we developed the DE method, we coupled it with sodium dodecyl sulfate polyacrylamide gel electrophoresis (SDS-PAGE) for protein separation and purification, followed by in-gel digestion to generate digested peptides for downstream liquid chromatography-tandem mass spectrometry (LC-MS/MS) analysis. Since SDS-PAGE and in-gel digestion are time-consuming and laborious, we further simplified protein purification for digestion by optimizing a published bead-based single-pot, solid-phase enhanced sample preparation (SP3) method.^6,7^ We demonstrated improved GVP discovery with the DE and optimized SP3 workflow, which was not only faster than the previous in-gel digestion method but also required significantly less instrument time, depending on the number of gel slices processed. Additionally, this led to increased numbers of identified proteins and GVPs.^7^

In this work, we examine another potential means of enhancing the ability to identify GVPs by exploring the effects of other proteolytic enzymes. Trypsin is almost universally used to generate peptides from proteins for a variety of reasons, such as generating optimal sets of peptides for mass spectrometry via selective cleavage at the C-terminus of arginine and lysine residues. Since it is known that the use of multiple enzymes can enhance sequence coverage in proteomics studies^8^ and KRT and KRTAP proteins are the main components of hair proteome,^4^ we examined several alternative enzymes other than trypsin (keratinase, chymotrypsin, and Glu-C), alone or combined with trypsin, to explore their ability to expand the sequence coverage of hair KRTs and KRTAPs and to generate other identifiable peptides, including GVPs and confirm the previous findings in tryptic digests.

## Methods

### Human Hair Sample Preparation

We used hair shafts from a randomly selected Asian male donor (AM, 30 years old) to set up our methods. Hair samples were commercially obtained from BioreclamationIVT (LOT# BRH1363732) under the approval of the NIST Research Protections Office (RPO# MML-17–0016) on a yearly basis. We briefly washed hair shafts with 20% methanol and water and then dried and stored them at −20 °C (at −80 °C for long-term storage).

### Trypsin and Enzymes Other than Trypsin

Proteins were extracted from single 5 cm-long hair shafts (about 100 ug each) of the AM donor by the DE method,^5^ reduced (dithiothreitol), and alkylated (iodoacetamide) using the workflow previously published,^5^ followed by purification and digestion with the optimized SP3 method.^7^

Several different enzymes were examined under the following conditions in 200 uL of 25 mM ammonium bicarbonate:

(1) Trypsin and LysC (T, Promega, V5111 and V1671): Enzyme-to-protein ratio of 1:20 and an overnight 18 h incubation period at 37 °C.

(2) Chymotrypsin (C, Promega, V1061): 1:10 and 2 h incubation at room temperature.

(3) Glu-C (G, Promega, V1651): 1:10 and overnight 18 h incubation at 37 °C.

(4) Keratinase (K, Sigma, K4519): 1 Unit (1U):20 and 2 h incubation at 50 °C.

(5) Keratinase with trypsin/LysC (KT): K, 1U:20, 2 h incubation at 50 °C, heat inactivation for 10 min at 95 °C, followed by T, 1:20, and overnight 18 h incubation at 37 °C.

(6) Chymotrypsin with trypsin/LysC (CT): C, 1:10, 2 h incubation at room temperature, heat inactivation of C for 10 min at 95 °C, then added T, 1:20, and overnight 18 h incubation at 37 °C.

(7) Glu-C with trypsin/LysC (GT): G, 1:10, together with T, 1:20, and overnight 18 h incubation at 37 °C.

Three replicates (labeled as 1, 2, and 3) were performed for each protease and each combination.

Peptides were cleaned up with MonoSpin C18 columns (GL Sciences Inc., 5010–21701) for LC-MS/MS on an Orbitrap Fusion Lumos mass spectrometer.^5^

LC-MS/MS parameters: Digests were analyzed on a Thermo Dionex Ultimate 3000 LC with an Acclaim PepMap 100 C18 (75 μm × 25 cm, C18, 2 μm, 100 A) with a nanospray source connected to a Thermo Orbitrap Fusion Lumos mass spectrometer in the positive ion mode. Solvent A consisted of 0.1% formic acid in water, and Solvent B consisted of 0.1% formic acid in acetonitrile. The peptides were eluted by increasing Solvent B using the following gradient over 230 min (min:B%): 0:2, 25:2, 142:32, 159:80, 165:98, 175:98, 180:2, and 230:2. Data were collected using a data-dependent mode with a dynamic exclusion of 20 s. MS/MS HCD fragmentation spectra were acquired with a cycle time of 4 s for precursor ions selected from 350 to 1600 *m*/*z* full survey scans. The resolution of the full MS scan was set at 120,000, and the resolution of the MS/MS scan was set at 30,000.

### Expanded FASTA File

The human protein FASTA file (UniProtKB/SwissProt, downloaded from NCBI, January 2022) with 20,375 sequences was expanded by adding mutations reported by BioMuta^9^ for 54 KRTs with 7554 proposed mutations and 92 KRTAPs with 4,534 proposed mutations. These mutations are either genetic or disease-related variants. For each mutation, a separate sequence entry was added to the FASTA file. For that purpose, we first used the reference protein sequence containing the mutation site and trimmed it to retain up to 40 flanking amino acids on both sides of the site. For a mutation site closer to the protein N- or C-terminus, the flanking amino acids could be less than 40. We then replaced the mutation site with the mutant (or variant) amino acids. Thus, most mutant sequence entries were 81 amino acids in length, with the variant residue occupying the center position. An identified peptide was considered a GVP only if it was mapped to a mutant sequence and contained a mutant amino acid.

### Sequence Database Searching

We used Sequest^10^ (Proteome Discoverer 2.4) for database searching with a precursor ion tolerance of 20 ppm and fragment ion tolerance of 50 ppm. The top-scoring peptide identification was selected, and the false discovery rate level was set at 1% (in the “Target-Decoy PSM Validator” node) using the FASTA file described above. Settings for modifications are (1) dynamic modification: oxidation on methionine and (2) static modification: carbamidomethyl on cysteine. Settings for enzymes are (1) full enzymatic searching for T, C, G, CT, and GT and (2) nonspecific searching for K and KT.

### Applied GVP Filters

GVP identifications were filtered using the following criteria: (1) the GVP sequence is not in the reference sequences; (2) the precursor ion tolerance of GVP was set up to 5 ppm to accurately distinguish glutamine and lysine; (3) the GVP sequence cannot be accounted for by a known common chemical modification in amino acid residues including isoleucine/leucine, asparagine/aspartic acid, and glutamine/glutamic acid; and (4) the length of the GVP was set to 7 to 40 amino acids.

## Results and Discussion

### Optimizing the Experimental Conditions of Keratinase

Keratinase, since it is not a highly specific protease, is not generally used in proteomic studies. However, it was selected for use in the present study, since keratins and keratin-associated proteins are the main components of the hair proteome. It was surmised that keratinase could loosen or weaken the mechanically strong structure of human hair, making it more accessible to trypsin. To find an optimal way to use keratinase, we first conducted a series of experiments.

For optimizing the usage of keratinase alone, we first used it at 1U:20 dilution by mass at 50 °C following the manufacturer’s instructions. Next, we tested two incubation conditions to find the optimal incubation period. One was the 2 h incubation as we described in the Methodssection (K), and another was an overnight 18 h incubation. We found significantly less GVPs from the overnight 18 h incubation (GVPs from 2 h: 31 ± 6; from 18 h: 10 ± 3). The potential reason might be due to the overdigestion of hair samples by the keratinase with longer incubation that generated the shorter peptides (<7 amino acids), which will be excluded by the downstream searching and data processing. Thus, we concluded that a 2 h incubation period for keratinase (1U:20) at 50 °C was the optimal experimental condition for the application of keratinase to the hair samples (K).

We also examined the usage of keratinase with trypsin. For testing the optimal enzymatic combination, besides the sequential combination of keratinase and trypsin/LysC as we described in the Methods section (KT), we also put keratinase (1U:20) together with trypsin/LysC for a 2 h or an overnight 18 h incubation period to test the enzymatic performance if these enzymes were added at the same time. Since the optimal temperature was different for keratinase (50 °C) versus trypsin/LysC (37 °C), one specific temperature had to be selected. We used 50 °C for the combined 2 h incubation (considering that 2 h was an ideal incubation period for keratinase from the previous discussion) and 37 °C for the combined 18 h incubation (considering that 18 h was a classic incubation period for trypsin/LysC). The average number of the identified GVPs declined from 54 to 11 with the extension of the incubation period from 2 to 18 h. Both numbers (54 ± 31 and 11 ± 3) were significantly lower than the average number of GVPs in the KT group (140 ± 17). The potential reason might be because K and T interfere with each other (particularly worse in the longer incubation period, K might have been mostly digested by T), resulting in poor enzymatic performance in the hair digests. On the other hand, if we used keratinase for a brief pretreatment under its optimal condition (1U:20, 50 °C, 2 h) and then deactivated it before adding trypsin/LysC for a classic tryptic digestion that was also under the optimal conditions (1:20, 37 °C, overnight 18 h), we achieved the best enzymatic performance of keratinase and trypsin/LysC in the hair digests (KT).

Since we obtained the best results from K and KT, we kept these experimental conditions (as K and KT) in the downstream sections in Results and Discussion.

### Enzyme Comparison for Hair Proteins, KRTs, and KRTAPs

Trypsin is typically the default enzyme for proteomic digestion.^1–5^ Here, we examined trypsin/LysC (T) as well as several other alternative enzymes including chymotrypsin (C) and Glu-C (G) as other specific enzymes and keratinase (K) as a nonspecific enzyme to explore their ability to generate both overlapping and additional identifiable regular or variant peptides from the hair proteome. All these nontrypsin enzymes belong to the different families of serine proteases with varying preferences as follows: (1) C tends to cleave at the C-terminus of tyrosine, phenylalanine, tryptophan, and leucine; (2) G tends to cleave at the C-terminus of either aspartic or glutamic acid residues; and (3) K is a nonspecific protease that cleaves nonterminal peptide bonds.^11^

We first compared T to C, G, and K for the identified hair proteins, KRTs, KRTAPs, and GVPs. As shown in Table 1, T is still the best single enzyme due to finding the most hair proteins, hair keratin-related proteins (including cuticular KRTs, cytoskeleton KRTs, and KRTAPs), hair cuticular KRTs, and hair GVPs, from the hair proteome using the AM donor’s single 5 cm-long hair strand as the starting material for each experiment. Detailed lists of GVPs identified by each enzyme are provided in Supporting Table S1. For KRTAPs, K found more KRTAPs (average by T: 18, C: 3, G: 0, and K: 21) with more corresponding peptides/spectral counts (PSMs) (average by T: 35/154, C: 4/9, G: 0/0, and K: 388/1,039). The result was expected since KRTAPs include high sulfur proteins with 20% cysteine residues and ultrahigh sulfur proteins with 30% to 40% cysteine residues;^12^ thus, the specific enzymes such as T, C, and G will not work efficiently because they lack their specific cleavage sites. On the other hand, keratinase, as a nonspecific enzyme,^11^ is not limited to any specific cleavage sites. Therefore, keratinase was expected to digest KRTAPs better than the tested specific enzymes.

**Table 1:** Enzyme Comparison of Human Hair Proteins, KRTs, KRTAPs, and GVPs^a^

	hair proteins			hair keratin-related			cuticular KRTs			KRTAPs			
E	proteins	peptides	PSMs	proteins	peptides	PSMs	proteins	peptides	PSMs	proteins	peptides	PSMs	GVPs^a^
T/C/G, Enzymatic													
T1	131	698	8059	42	561	7859	13	447	7526	17	34	155	64
T2	120	656	6855	45	552	6681	12	405	6238	20	38	159	55
T3	109	686	8911	39	573	8726	13	468	8424	16	33	147	64
C1	81	291	1228	21	186	980	11	164	926	3	4	8	10
C2	66	185	582	16	106	407	7	89	378	4	7	13	8
C3	77	261	1075	19	174	887	12	161	859	2	2	6	10
G1	69	283	2852	15	204	2724	9	181	2378	0	0	0	20
G2	86	407	4623	19	294	4423	11	254	3926	0	0	0	24
G3	92	394	4487	18	271	4264	11	239	3770	0	0	0	25
K, Unspecific													
K1	55	694	1904	34	660	1827	7	175	450	21	403	1216	35
K2	49	572	1745	34	550	1706	8	213	673	21	300	945	24
K3	50	822	2452	34	801	2414	8	334	1447	22	461	955	35
KT, Unspecific													
KT1	127	2114	12,403	56	2023	12,267	15	856	7381	32	1110	4677	159
KT2	155	1885	19,142	52	1741	18,917	12	1012	16,124	30	669	2579	129
KT3	152	1888	16,652	59	1762	16,448	15	990	14,047	33	689	2107	132
CT/GT, Enzymatic													
CT1	111	343	4936	21	223	4735	11	191	4489	1	1	21	16
CT2	121	432	7688	23	305	7469	13	257	7142	1	1	21	14
CT3	105	324	4879	20	208	4683	9	157	4316	1	1	19	14
GT1	128	451	2154	20	208	1649	12	185	1580	1	1	5	7
GT2	131	434	2259	21	196	1806	12	170	1745	2	2	4	9
GT3	147	513	2703	24	244	2155	13	215	2077	3	3	9	15

a The detailed GVP list is shown in Supporting Information Table S1. Three replicates (labeled as 1, 2, and 3) were performed for each protease (T, C, G, and K) and combination (KT, CT, and GT).

We then tested these alternative enzymes (C, G, and K) combined with T (KT, CT, and GT) for the identified hair proteins, KRTs, KRTAPs, and GVPs (Table 1). From these different enzymatic combination studies, it was observed that KT performed the best, identifying the most hair proteins, hair keratin-related proteins, hair cuticular KRTs, KRTAPs, and GVPs. The KT group (140) performed better than T or K alone by summing the GVP results of independent digestions (61­(by T) + 31­(by K) = 92). Furthermore, we identified approximately two times as many peptides in the KT group when compared to T or K alone (average peptides/PSMs for T, K, and KT: 680/7 942, 696/2034, and 1962/16,066). These results suggested that the addition of keratinase pretreatment facilitated the downstream trypsin to digest hair proteins in a more efficient way, possibly by making them more accessible to trypsin. Detailed lists of identified GVPs from these different enzymatic combination studies can be found in Supporting Information Table S1.

### Complementary Sequence Coverage by Different Enzymes

It is known that multiple enzymatic digestions^8^ will enhance the sequence coverage in proteomic studies. From the above section, Table 1 clearly shows that trypsin remained the single best enzyme, but the introduction of alternative proteases expanded the sequence coverage of the identified hair proteins and therefore increased the likelihood of identifying regular/variant peptides, in addition to confirming the findings from the tryptic digests. Here, we examined several single enzymes (T, C, G, and K) as well as their combined groups (KT, CT, and GT) to explore their ability to generate both overlapping and additional identifiable peptides to expand the sequence coverage of the identified hair proteins (mainly KRTs and KRTAPs).

#### KRTs

Figure 1 shows the complementary sequence coverage of an example hair type I cuticular KRT (GN = KRT31), and Figure 2 shows the complementary sequence coverage of an example hair type II cuticular KRT (GN = KRT81) by three different enzymatic digestions (T, K, and KT). A single 5 cm-long hair strand from the same AM donor was used as the starting material in each experiment. The identified reference sequences are highlighted in green. The sequence coverage of KRT31 by T, K, and KT was 81%, 32%, and 96%, respectively, and the sequence coverage of KRT81 by T, K, and KT was 76%, 39%, and 83%, respectively. Both Figures 1 and 2 illustrate the complementary nature of T and K. The combined KT group expanded the sequence coverage of two example major hair cuticular keratins (for KRT31: from 81% to 96%; for KRT81: from 76% to 83%) by increasing about 10% per each.

**Figure 1:**
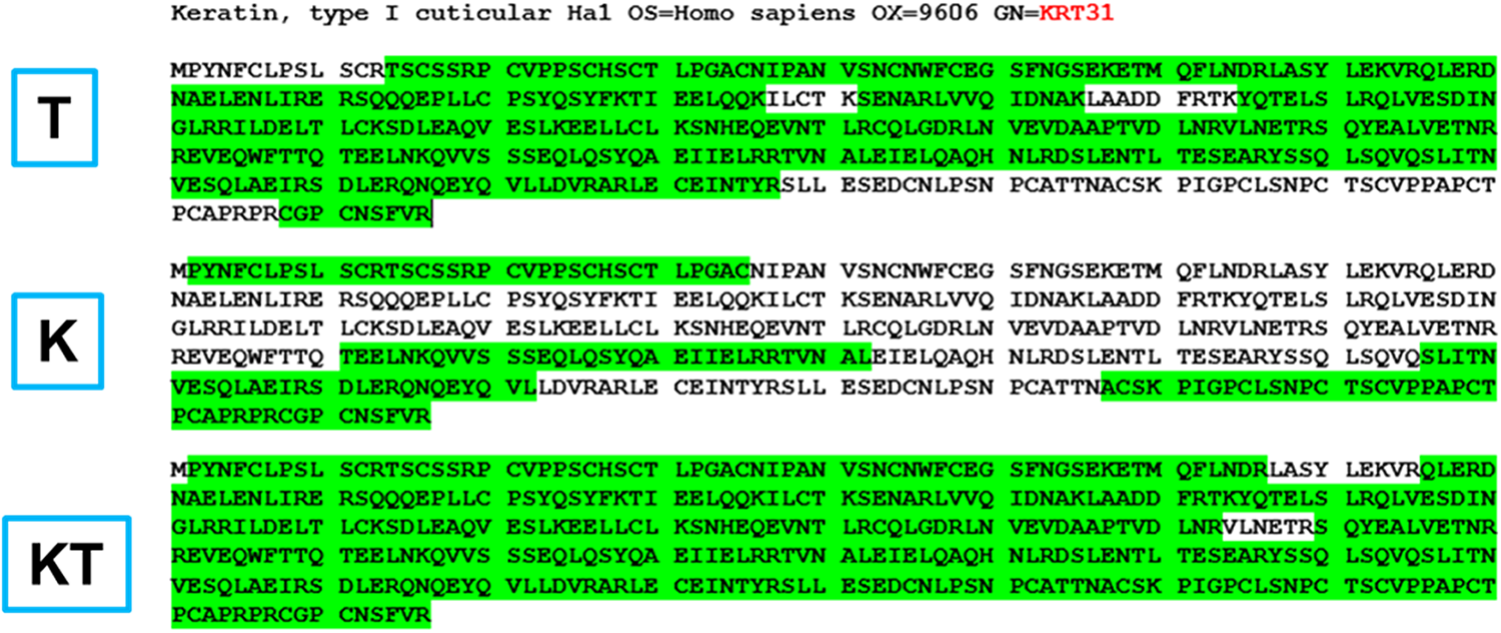
Complementary sequence coverage of an example hair type I cuticular KRT31 (sp|Q15323|K1H1_HUMAN Keratin, type I cuticular Ha1 OS = *Homo sapiens*) by different enzyme digestion studies (T, K, and KT) on hair samples from the same donor. An individual 5 cm-long hair strand was used as the starting material in each experiment. The identified reference sequences are highlighted in green. Sequence coverage of KRT31 by T, K, and KT: 81%, 32%, and 96%, respectively. The sequence coverage of KRT31 from each single/combined enzyme study can be found in Supporting Figure S1.

**Figure 2:**
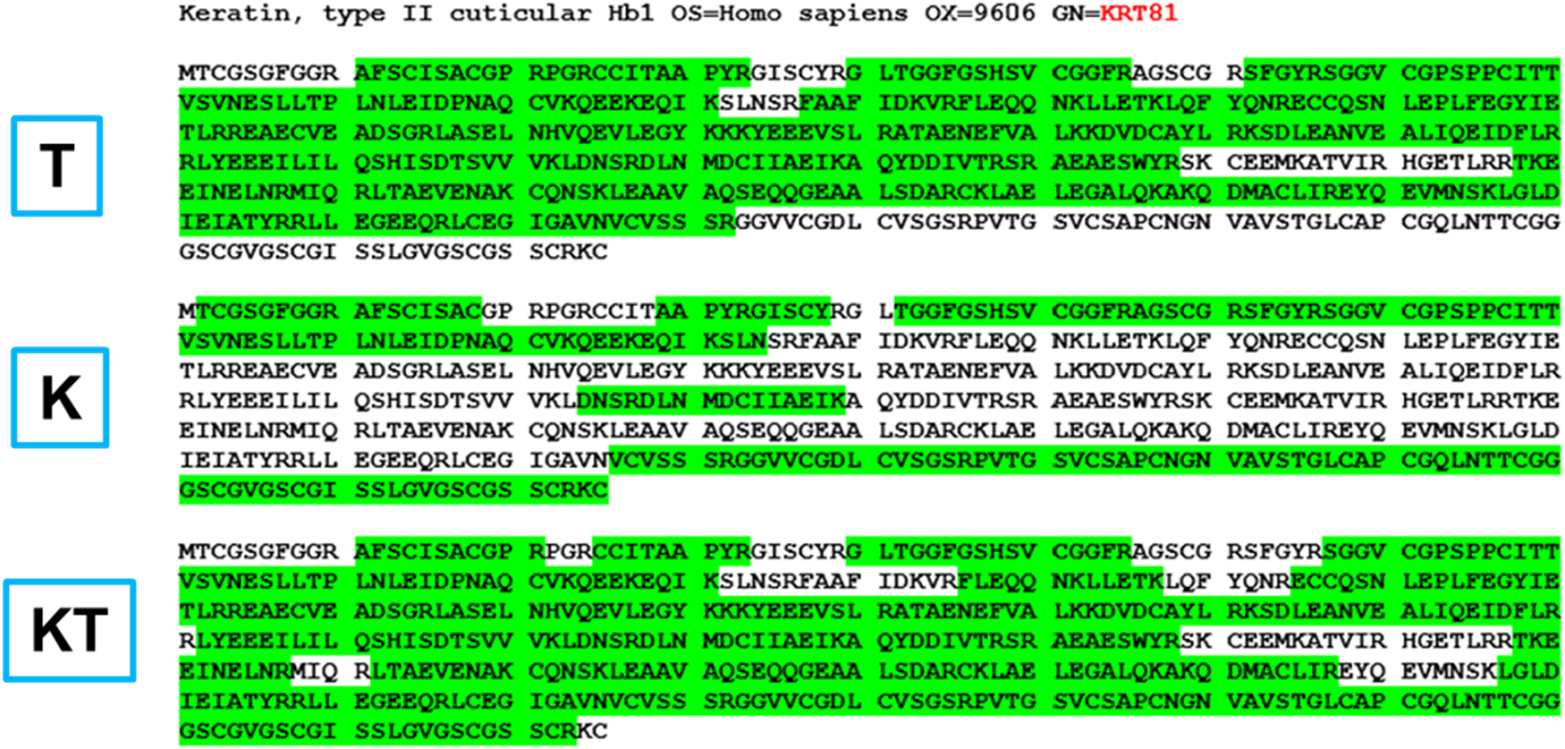
Complementary sequence coverage of an example hair type II cuticular KRT81 (sp|Q14533|KRT81_HUMAN Keratin, type II cuticular Hb1 OS = *Homo sapiens*) by different enzyme digestion studies (T, K, and KT) on hair samples from the same donor. An individual 5 cm-long hair strand was used as the starting material in each experiment. The identified reference sequences were highlighted in green. Sequence coverage of KRT81 by T, K, and KT: 76%, 39%, and 83%, respectively. The sequence coverage of KRT81 from each single/combined enzyme study can be found in Supporting Figure S1.

A more comprehensive view of the sequence coverage of KRT31 and KRT81 can be found in Supporting Information Figure S1, which lists the sequence coverage of KRT31 and KRT81, from all single enzyme experiments (T, C, G, and K), as well as all combined experiments (KT, CT, and GT) if KRT31 or KRT81 was identified in that experiment. Supporting Information Figure S1 clearly indicates the complementary nature of these various enzymes, and the combined group, including KT, enhanced the sequence coverage of both KRT31 and KRT81.

#### KRTAPs

The KT group also showed the highest sequence coverage (average of 64.7%) for the identified KRTAPs, better than the results obtained from the other enzymatic digestions (average for T, C, G, K, CT, and GT: 27.8%, 11.5%, 0%, 47.2%, 7%, and 12.2%). It was not surprising that the sequence coverage for the KRTAPs using K alone reached 47.2% as this was consistent with the results shown in Table 1. As we discussed before, keratinase, as a nonspecific enzyme,^11^ was expected to digest KRTAPs more efficiently than the specific enzymes tested in this manuscript. Thus, introducing a brief keratinase pretreatment followed by trypsin digestion of the hair samples increases the sequence coverage percentiles for both KRTs and KRTAPs.

### Enhanced GVP Profiling from Human Hair

Introducing enzymes other than trypsin has another advantage: it improves the identification of variant peptides and sites. Let us look at two examples, as shown in Tables 2 and 3.

**Table 2:**
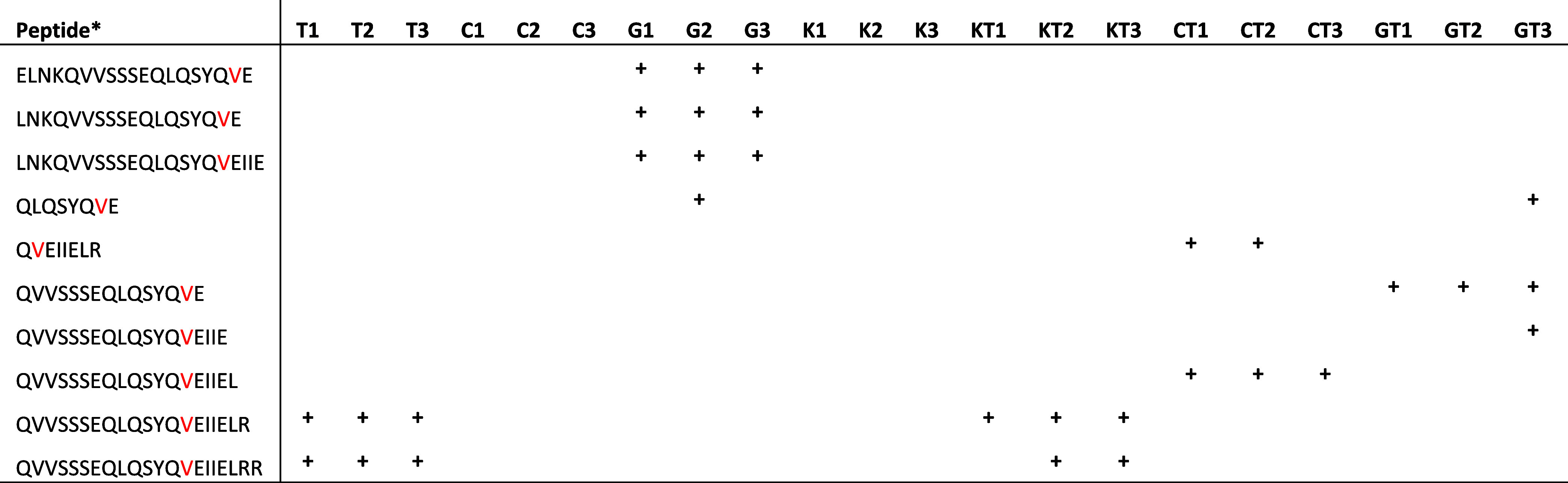
Example Variant Site A270 V in KRT31 Identified by Different Peptides Generated by Various Enzymes^a^

a The mutated amino acid ‘V’ is highlighted in red. “+” indicates that the listed peptide was detected.

**Table 3:**
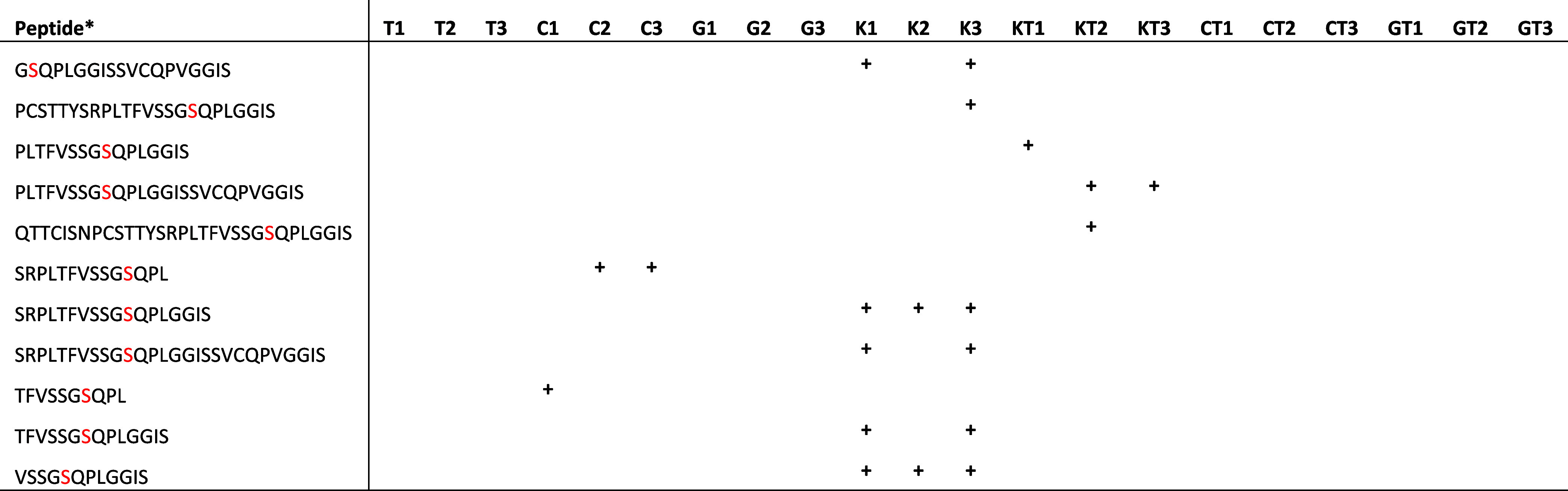
Example Variant Site C111S in KRTAP11-1 Identified by Different Peptides Generated by Various Enzymes^a^

a The mutated amino acid ‘S’ is highlighted in red. “+” indicates that the listed peptide was detected.

Table 2 shows the identification of an example single amino acid polymorphism (SAP) site called KRT31_A270 V. At amino acid position 270 of type I cuticular keratin 31, alanine was changed to valine (valine highlighted as “V” in red in Table 2). From the peptide column listed in Table 2, besides the two tryptic peptides identified by T and KT, there are several other peptides, like the three generated by G, one by both G and GT, two by CT, and two by GT, suggesting the identification of the same variant SAP site. The fact that this SAP site was identified by the overlapping GVP sequences generated by the various enzymatic digestions greatly increased the confidence of the identification of this variant site.

Furthermore, each enzyme identified unique variant peptides representing variant sites that could be missed by trypsin, such as the SAP site KRTAP11–1_C111S shown in Table 3. At amino acid position 111 of keratin-associated protein 11–1, cysteine was changed to serine. Serine “S” is highlighted in red. An improved discovery of this SAP site was achieved by introducing the alternative enzymatic digestions from C, K, and KT. From the peptide column listed in Table 3, we observed that none of these variant peptides were generated by T. Instead, two were generated by C, six peptides were generated by K, and three peptides were generated by KT. The fact that multiple variant peptides were identified via the different enzymes (C and K) or the combined group (KT) indicated the complementary nature of these nontrypsin enzymes and their unique capability for the identification of GVPs and SAPs that were missed by trypsin.

A combined GVP panel, as shown in Supporting Information Table S1, listing all GVPs identified and filtered by the various enzymatic digestions (T, C, G, K, KT, CT, and GT), can be used to conveniently compare the results generated by these digestions. Figure 3, as an example, shows a Venn diagram to compare the identification of GVPs found by three different enzymatic digestions (T, K, and KT): (1) T (102), the best of the single enzyme group; (2) K (62), the key nonspecific enzyme that we introduced for the hair keratins; and (3) KT (286), the best of the combined group and the best of all tested conditions in this manuscript. The large number of unique GVP identifications (235) found by the KT group was encouraging as the more GVPs we find, the better they can serve as a distinguishing tool for human identification. It confirmed our hypothesis that introducing keratinase will help trypsin in tough tissues including hair, considering that KRTs and KRTAPs are the main components of the hair proteome. It was notable that a good proportion of tryptic findings could be confirmed by the combined KT group (48 out of 102). On the other hand, the overlap between T and K or between KT and K was small (4 between T and K; 7 between KT and K), suggesting the complementary nature of T and K.

**Figure 3:**
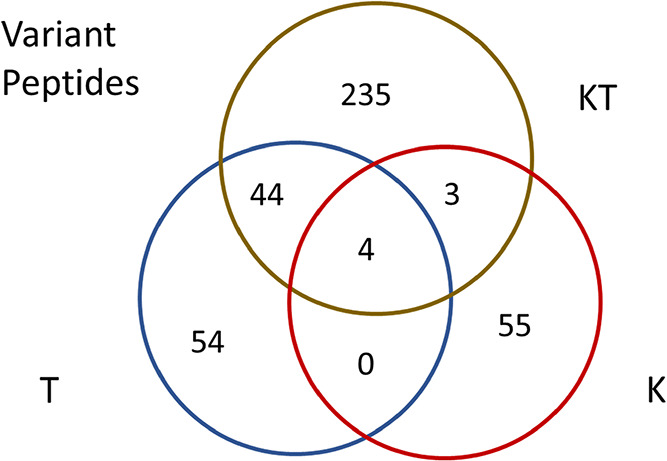
Venn Diagram to show the identification of variant peptides by different enzymes. KT represents the variant peptides found by the combined KT group; T represents the variant peptides found by T; K represents the variant peptides found by K. The detailed variant peptide list with the mutated amino acid highlighted in red from each single or combined enzymatic experiment can be found in Supporting Table S1.

Several observations are summarized here by going through the combined GVP panel as shown in Supporting Information Table S1: (1) we found a total of 472 GVPs, representing 279 SAP sites, from hair KRTs and KRTAPs when we combined all the experiments (T, C, G, K, KT, CT, and GT); (2) about 30% SAP sites were identified by two or more variant peptides from the same or different experiment(s); (3) for the 102 GVPs found by just T, 49 (48%) can also be confirmed by other digestions either using nontrypsin enzyme alone (4 by K) or combined with trypsin (48 by KT and 6 by CT); and (4) each nontrypsin enzyme (K/C/G) also made the unique contribution in identifying its own GVPs (K: 55, C: 15, G: 34). Putting all of these together, introducing multiple enzymes for hair protein digestion enhanced GVP profiling from human hair samples.

## Summary and Conclusions

The direct extraction method^5^ simplifies protein extraction from hair samples, and the optimized SP3 method^7^ further simplifies sample purification for enzyme digestion. These simplified and optimized sample preparation procedures enable us to perform proteomic experiments more efficiently and conveniently, including the comprehensive multiple enzyme digestions we tested in this manuscript. Enzymes other than trypsin expanded the sequence coverage in hair keratins and keratin-associated proteins, therefore increasing the chance of a regular or variant peptide being identified, as well as confirming the findings from the tryptic digests. Overall, we presented an enhanced variant peptide discovery workflow by coupling alternative enzymes with trypsin, resulting in superior identification of variant peptides and variant amino acid polymorphism sites in the human hair samples we tested. Presumably, the finding may have general applicability to other donors’ hair samples or other types of samples, such as human skin, which we would like to explore in our future studies. It is also conceivable that mass spectrometry-based proteomic methods, when combined with personalized next-generation genomic sequencing, could significantly speed up the discovery and validation of GVPs^13^ that may one day be used in the real world to identify victims and criminals when DNA evidence is lacking.

## Supplementary Material





## Data Availability

All raw data files are available on MassIVE (MSV000101400, password: 9DQ3u0utslxhA9GR).

## Notes

Sequence coverage of KRT31 and KRT81 from each study (PDF)
Comparison of the identified GVPs for various enzyme digestions (XLSX)

## Abbreviations

NIST: National Institute of Standards and Technology
NCBI: National Center for Biotechnology Information
RPO: Research Protections Office
DE: direct extraction
SDS: sodium dodecyl sulfate
SDS-PAGE: sodium dodecyl sulfate polyacrylamide gel electrophoresis
LC-MS/MS: liquid chromatography-tandem mass spectrometry
SP3: single-pot, solid-phase-enhanced sample preparation
AM: Asian male donor
GN: gene Name
KRT: keratin
KRTAP: keratin-associated protein
PSM: peptide-spectrum match
SAP: single amino acid polymorphism
GVP: genetically Variant Peptide
FASTA: a text-based format for representing protein sequences
T: trypsin/LysC
C: chymotrypsin
G: Glu-C
K: keratinase
KT: keratinase with trypsin/LysC
CT: chymotrypsin with trypsin/LysC
GT: Glu-C with trypsin/LysC
