# Supplementary material for: Enhanced Genetically Variant Peptide Profiling from Human Hair Using Multiple Enzymes
Source: ACS Omega. 2026 Jun 29;11(27):40711–7. doi: 10.1021/acsomega.6c04197 (PMC13382710; doi:10.1021/acsomega.6c04197)
Supplement: Supplementary file 1 [file ao6c04197_si_001.pdf]

Enhanced Genetically Variant Peptide Profiling from Human Hair Using Multiple Enzymes

Zheng Zhang[1]*, William E. Wallace[1], Guanghui Wang[1], Meghan C. Burke[1], Stephen E. Stein[1]

[1]Mass Spectrometry Data Center, Biomolecular Measurement Division, National Institute of Standards and Technology, 100 Bureau Drive, Gaithersburg, Maryland 20899 USA

Correspondence to:

*E-mail: zheng.zhang@nist.gov Tel: +1 301-975-5828

Sequence coverage of KRT31 from each study.

GN=KRT31: Keratin, type I cuticular Ha1 OS=Homo sapiens

T1 81.49%

```
MPYNFCLPSL SCRTSCSSRP CVPPSCHSCT LPGACNIPAN VSNCNWFCEG SFNGSEKETM QFLNDRLASY LEKVRQLERD
NAELENLIRE RSQQQEPLLC PSYQSYFKTI EELQQKILCT KSENARLVVQ IDNAKLAADD FRTKYQTELS LRQLVESDIN
GLRRILDELT LCKSDLEAQV ESLKEELLCL KSNHEQEVNT LRCQLGDRLN VEVDAAPTVD LNRVLNETRS QYEALVETNR
REVEQWFTTQ TEELNKQVVS SSEQLQSYQA EIIELRRTVN ALEIELQAQH NLRDSLENTL TESEARYSSQ LSQVQSLITN
VESQLAEIRS DLERQNQEYQ VLLDVRARLE CEINTYRSLL ESEDCNLPSN PCATTNACSK PIGPCLSNPC TSCVPPAPCT
PCAPRPRCGP CNSFVR
```

T2 84.38%

```
MPYNFCLPSL SCRTSCSSRP CVPPSCHSCT LPGACNIPAN VSNCNWFCEG SFNGSEKETM QFLNDRLASY LEKVRQLERD
NAELENLIRE RSQQQEPLLC PSYQSYFKTI EELQQKILCT KSENARLVVQ IDNAKLAADD FRTKYQTELS LRQLVESDIN
GLRRILDELT LCKSDLEAQV ESLKEELLCL KSNHEQEVNT LRCQLGDRLN VEVDAAPTVD LNRVLNETRS QYEALVETNR
REVEQWFTTQ TEELNKQVVS SSEQLQSYQA EIIELRRTVN ALEIELQAQH NLRDSLENTL TESEARYSSQ LSQVQSLITN
VESQLAEIRS DLERQNQEYQ VLLDVRARLE CEINTYRSLL ESEDCNLPSN PCATTNACSK PIGPCLSNPC TSCVPPAPCT
PCAPRPRCGP CNSFVR
```

T3 83.17%

```
MPYNFCLPSL SCRTSCSSRP CVPPSCHSCT LPGACNIPAN VSNCNWFCEG SFNGSEKETM QFLNDRLASY LEKVRQLERD
NAELENLIRE RSQQQEPLLC PSYQSYFKTI EELQQKILCT KSENARLVVQ IDNAKLAADD FRTKYQTELS LRQLVESDIN
GLRRILDELT LCKSDLEAQV ESLKEELLCL KSNHEQEVNT LRCQLGDRLN VEVDAAPTVD LNRVLNETRS QYEALVETNR
REVEQWFTTQ TEELNKQVVS SSEQLQSYQA EIIELRRTVN ALEIELQAQH NLRDSLENTL TESEARYSSQ LSQVQSLITN
VESQLAEIRS DLERQNQEYQ VLLDVRARLE CEINTYRSLL ESEDCNLPSN PCATTNACSK PIGPCLSNPC TSCVPPAPCT
PCAPRPRCGP CNSFVR
```

K1 31.97%

```
MPYNFCLPSL SCRTSCSSRP CVPPSCHSCT LPGACNIPAN VSNCNWFCEG SFNGSEKETM QFLNDRLASY LEKVRQLERD
NAELENLIRE RSQQQEPLLC PSYQSYFKTI EELQQKILCT KSENARLVVQ IDNAKLAADD FRTKYQTELS LRQLVESDIN
GLRRILDELT LCKSDLEAQV ESLKEELLCL KSNHEQEVNT LRCQLGDRLN VEVDAAPTVD LNRVLNETRS QYEALVETNR
REVEQWFTTQ TEELNKQVVS SSEQLQSYQA EIIELRRTVN ALEIELQAQH NLRDSLENTL TESEARYSSQ LSQVQSLITN
VESQLAEIRS DLERQNQEYQ VLLDVRARLE CEINTYRSLL ESEDCNLPSN PCATTNACSK PIGPCLSNPC TSCVPPAPCT
PCAPRPRCGP CNSFVR
```

K2 36.06%

```
MPYNFCLPSL SCRTSCSSRP CVPPSCHSCT LPGACNIPAN VSNCNWFCEG SFNGSEKETM QFLNDRLASY LEKVRQLERD
NAELENLIRE RSQQQEPLLC PSYQSYFKTI EELQQKILCT KSENARLVVQ IDNAKLAADD FRTKYQTELS LRQLVESDIN
GLRRILDELT LCKSDLEAQV ESLKEELLCL KSNHEQEVNT LRCQLGDRLN VEVDAAPTVD LNRVLNETRS QYEALVETNR
REVEQWFTTQ TEELNKQVVS SSEQLQSYQA EIIELRRTVN ALEIELQAQH NLRDSLENTL TESEARYSSQ LSQVQSLITN
VESQLAEIRS DLERQNQEYQ VLLDVRARLE CEINTYRSLL ESEDCNLPSN PCATTNACSK PIGPCLSNPC TSCVPPAPCT
PCAPRPRCGP CNSFVR
```

K3 71.63%

```
MPYNFCLPSL SCRTSCSSRP CVPPSCHSCT LPGACNIPAN VSNCNWFCEG SFNGSEKETM QFLNDRLASY LEKVRQLERD
NAELENLIRE RSQQQEPLLC PSYQSYFKTI EELQQKILCT KSENARLVVQ IDNAKLAADD FRTKYQTELS LRQLVESDIN
GLRRILDELT LCKSDLEAQV ESLKEELLCL KSNHEQEVNT LRCQLGDRLN VEVDAAPTVD LNRVLNETRS QYEALVETNR
REVEQWFTTQ TEELNKQVVS SSEQLQSYQA EIIELRRTVN ALEIELQAQH NLRDSLENTL TESEARYSSQ LSQVQSLITN
VESQLAEIRS DLERQNQEYQ VLLDVRARLE CEINTYRSLL ESEDCNLPSN PCATTNACSK PIGPCLSNPC TSCVPPAPCT
PCAPRPRCGP CNSFVR
```

KT1 96.15%

MPYNFCLPSL SCRTSCSSRP CVPPSCHSCT LPGACNIPAN VSNCNWFCEG SFNGSEKETM QFLNDRLASY LEKVRQLERD
NAELENLIRE RSQQQEPLLC PSYQSYFKTI EELQQKILCT KSENARLVVQ IDNAKLAADD FRTKYQTELS LRQLVESDIN
GLRRILDELT LCKSDLEAQV ESLKEELLCL KSNHEQEVNT LRCQLGDRLN VEVDAAPTVD LNRVLNETRS QYEALVETNR
REVEQWFTTQ TEELNKQVVS SSEQLQSYQA EIIELRRTVN ALEIELQAQH NLRDSLENTL TESEARYSSQ LSQVQSLITN
VESQLAEIRS DLERQNQEYQ VLLDVRARLE CEINTYRSLL ESEDCNLPSN PCATTNACSK PIGPCLSNPC TSCVPPAPCT
PCAPRPRCGP CNSFVR

KT2 95.43%

MPYNFCLPSL SCRTSCSSRP CVPPSCHSCT LPGACNIPAN VSNCNWFCEG SFNGSEKETM QFLNDRLASY LEKVRQLERD
NAELENLIRE RSQQQEPLLC PSYQSYFKTI EELQQKILCT KSENARLVVQ IDNAKLAADD FRTKYQTELS LRQLVESDIN
GLRRILDELT LCKSDLEAQV ESLKEELLCL KSNHEQEVNT LRCQLGDRLN VEVDAAPTVD LNRVLNETRS QYEALVETNR
REVEQWFTTQ TEELNKQVVS SSEQLQSYQA EIIELRRTVN ALEIELQAQH NLRDSLENTL TESEARYSSQ LSQVQSLITN
VESQLAEIRS DLERQNQEYQ VLLDVRARLE CEINTYRSLL ESEDCNLPSN PCATTNACSK PIGPCLSNPC TSCVPPAPCT
PCAPRPRCGP CNSFVR

KT3 95.43%

MPYNFCLPSL SCRTSCSSRP CVPPSCHSCT LPGACNIPAN VSNCNWFCEG SFNGSEKETM QFLNDRLASY LEKVRQLERD
NAELENLIRE RSQQQEPLLC PSYQSYFKTI EELQQKILCT KSENARLVVQ IDNAKLAADD FRTKYQTELS LRQLVESDIN
GLRRILDELT LCKSDLEAQV ESLKEELLCL KSNHEQEVNT LRCQLGDRLN VEVDAAPTVD LNRVLNETRS QYEALVETNR
REVEQWFTTQ TEELNKQVVS SSEQLQSYQA EIIELRRTVN ALEIELQAQH NLRDSLENTL TESEARYSSQ LSQVQSLITN
VESQLAEIRS DLERQNQEYQ VLLDVRARLE CEINTYRSLL ESEDCNLPSN PCATTNACSK PIGPCLSNPC TSCVPPAPCT
PCAPRPRCGP CNSFVR

C1 37.98%

MPYNFCLPSL SCRTSCSSRP CVPPSCHSCT LPGACNIPAN VSNCNWFCEG SFNGSEKETM QFLNDRLASY LEKVRQLERD
NAELENLIRE RSQQQEPLLC PSYQSYFKTI EELQQKILCT KSENARLVVQ IDNAKLAADD FRTKYQTELS LRQLVESDIN
GLRRILDELT LCKSDLEAQV ESLKEELLCL KSNHEQEVNT LRCQLGDRLN VEVDAAPTVD LNRVLNETRS QYEALVETNR
REVEQWFTTQ TEELNKQVVS SSEQLQSYQA EIIELRRTVN ALEIELQAQH NLRDSLENTL TESEARYSSQ LSQVQSLITN
VESQLAEIRS DLERQNQEYQ VLLDVRARLE CEINTYRSLL ESEDCNLPSN PCATTNACSK PIGPCLSNPC TSCVPPAPCT
PCAPRPRCGP CNSFVR

C2 34.62%

MPYNFCLPSL SCRTSCSSRP CVPPSCHSCT LPGACNIPAN VSNCNWFCEG SFNGSEKETM QFLNDRLASY LEKVRQLERD
NAELENLIRE RSQQQEPLLC PSYQSYFKTI EELQQKILCT KSENARLVVQ IDNAKLAADD FRTKYQTELS LRQLVESDIN
GLRRILDELT LCKSDLEAQV ESLKEELLCL KSNHEQEVNT LRCQLGDRLN VEVDAAPTVD LNRVLNETRS QYEALVETNR
REVEQWFTTQ TEELNKQVVS SSEQLQSYQA EIIELRRTVN ALEIELQAQH NLRDSLENTL TESEARYSSQ LSQVQSLITN
VESQLAEIRS DLERQNQEYQ VLLDVRARLE CEINTYRSLL ESEDCNLPSN PCATTNACSK PIGPCLSNPC TSCVPPAPCT
PCAPRPRCGP CNSFVR

C3 36.54%

MPYNFCLPSL SCRTSCSSRP CVPPSCHSCT LPGACNIPAN VSNCNWFCEG SFNGSEKETM QFLNDRLASY LEKVRQLERD
NAELENLIRE RSQQQEPLLC PSYQSYFKTI EELQQKILCT KSENARLVVQ IDNAKLAADD FRTKYQTELS LRQLVESDIN
GLRRILDELT LCKSDLEAQV ESLKEELLCL KSNHEQEVNT LRCQLGDRLN VEVDAAPTVD LNRVLNETRS QYEALVETNR
REVEQWFTTQ TEELNKQVVS SSEQLQSYQA EIIELRRTVN ALEIELQAQH NLRDSLENTL TESEARYSSQ LSQVQSLITN
VESQLAEIRS DLERQNQEYQ VLLDVRARLE CEINTYRSLL ESEDCNLPSN PCATTNACSK PIGPCLSNPC TSCVPPAPCT
PCAPRPRCGP CNSFVR

CT1 48.56%

MPYNFCLPSL SCRTSCSSRP CVPPSCHSCT LPGACNIPAN VSNCNWFCEG SFNGSEKETM QFLNDRLASY LEKVRQLERD
NAELENLIRE RSQQQEPLLC PSYQSYFKTI EELQQKILCT KSENARLVVQ IDNAKLAADD FRTKYQTELS LRQLVESDIN
GLRRILDELT LCKSDLEAQV ESLKEELLCL KSNHEQEVNT LRCQLGDRLN VEVDAAPTVD LNRVLNETRS QYEALVETNR
REVEQWFTTQ TEELNKQVVS SSEQLQSYQA EIIELRRTVN ALEIELQAQH NLRDSLENTL TESEARYSSQ LSQVQSLITN
VESQLAEIRS DLERQNQEYQ VLLDVRARLE CEINTYRSLL ESEDCNLPSN PCATTNACSK PIGPCLSNPC TSCVPPAPCT
PCAPRPRCGP CNSFVR

CT2 49.76%

MPYNFCLPSL SCRTSCSSRP CVPPSCHSCT LPGACNIPAN VSNCNWFCEG SFNGSEKETM QFLNDRLASY LEKVRQLERD
NAELENLIRE RSQQQEPLLC PSYQSYFKTI EELQQKILCT KSENARLVVQ IDNAKLAADD FRTKYQTELS LRQLVESDIN
GLRRILDELT LCKSDLEAQV ESLKEELLCL KSNHEQEVNT LRCQLGDRLN VEVDAAPTVD LNRVLNETRS QYEALVETNR
REVEQWFTTQ TEELNKQVVS SSEQLQSYQA EIIELRRTVN ALEIELQAQH NLRDSLENTL TESEARYSSQ LSQVQSLITN
VESQLAEIRS DLERQNQEYQ VLLDVRARLE CEINTYRSLL ESEDCNLPSN PCATTNACSK PIGPCLSNPC TSCVPPAPCT
PCAPRPRCGP CNSFVR

CT3 (no KRT31)

G1 51.44%

MPYNFCLPSL SCRTSCSSRP CVPPSCHSCT LPGACNIPAN VSNCNWFCEG SFNGSEKETM QFLNDRLASY LEKVRQLERD
NAELENLIRE RSQQQEPLLC PSYQSYFKTI EELQQKILCT KSENARLVVQ IDNAKLAADD FRTKYQTELS LRQLVESDIN
GLRRILDELT LCKSDLEAQV ESLKEELLCL KSNHEQEVNT LRCQLGDRLN VEVDAAPTVD LNRVLNETRS QYEALVETNR
REVEQWFTTQ TEELNKQVVS SSEQLQSYQA EIIELRRTVN ALEIELQAQH NLRDSLENTL TESEARYSSQ LSQVQSLITN
VESQLAEIRS DLERQNQEYQ VLLDVRARLE CEINTYRSLL ESEDCNLPSN PCATTNACSK PIGPCLSNPC TSCVPPAPCT
PCAPRPRCGP CNSFVR

G2 63.70%

MPYNFCLPSL SCRTSCSSRP CVPPSCHSCT LPGACNIPAN VSNCNWFCEG SFNGSEKETM QFLNDRLASY LEKVRQLERD
NAELENLIRE RSQQQEPLLC PSYQSYFKTI EELQQKILCT KSENARLVVQ IDNAKLAADD FRTKYQTELS LRQLVESDIN
GLRRILDELT LCKSDLEAQV ESLKEELLCL KSNHEQEVNT LRCQLGDRLN VEVDAAPTVD LNRVLNETRS QYEALVETNR
REVEQWFTTQ TEELNKQVVS SSEQLQSYQA EIIELRRTVN ALEIELQAQH NLRDSLENTL TESEARYSSQ LSQVQSLITN
VESQLAEIRS DLERQNQEYQ VLLDVRARLE CEINTYRSLL ESEDCNLPSN PCATTNACSK PIGPCLSNPC TSCVPPAPCT
PCAPRPRCGP CNSFVR

G3 57.93%

MPYNFCLPSL SCRTSCSSRP CVPPSCHSCT LPGACNIPAN VSNCNWFCEG SFNGSEKETM QFLNDRLASY LEKVRQLERD
NAELENLIRE RSQQQEPLLC PSYQSYFKTI EELQQKILCT KSENARLVVQ IDNAKLAADD FRTKYQTELS LRQLVESDIN
GLRRILDELT LCKSDLEAQV ESLKEELLCL KSNHEQEVNT LRCQLGDRLN VEVDAAPTVD LNRVLNETRS QYEALVETNR
REVEQWFTTQ TEELNKQVVS SSEQLQSYQA EIIELRRTVN ALEIELQAQH NLRDSLENTL TESEARYSSQ LSQVQSLITN
VESQLAEIRS DLERQNQEYQ VLLDVRARLE CEINTYRSLL ESEDCNLPSN PCATTNACSK PIGPCLSNPC TSCVPPAPCT
PCAPRPRCGP CNSFVR

GT1 42.07%

MPYNFCLPSL SCRTSCSSRP CVPPSCHSCT LPGACNIPAN VSNCNWFCEG SFNGSEKETM QFLNDRLASY LEKVRQLERD
NAELENLIRE RSQQQEPLLC PSYQSYFKTI EELQQKILCT KSENARLVVQ IDNAKLAADD FRTKYQTELS LRQLVESDIN
GLRRILDELT LCKSDLEAQV ESLKEELLCL KSNHEQEVNT LRCQLGDRLN VEVDAAPTVD LNRVLNETRS QYEALVETNR
REVEQWFTTQ TEELNKQVVS SSEQLQSYQA EIIELRRTVN ALEIELQAQH NLRDSLENTL TESEARYSSQ LSQVQSLITN
VESQLAEIRS DLERQNQEYQ VLLDVRARLE CEINTYRSLL ESEDCNLPSN PCATTNACSKPIGPCLSNPC TSCVPPAPCT
PCAPRPRCGP CNSFVR

GT2 38.46%

MPYNFCLPSL SCRTSCSSRP CVPPSCHSCT LPGACNIPAN VSNCNWFCEG SFNGSEKETM QFLNDRLASY LEKVRQLERD
NAELENLIRE RSQQQEPLLC PSYQSYFKTI EELQQKILCT KSENARLVVQ IDNAKLAADD FRTKYQTELS LRQLVESDIN
GLRRILDELT LCKSDLEAQV ESLKEELLCL KSNHEQEVNT LRCQLGDRLN VEVDAAPTVD LNRVLNETRS QYEALVETNR
REVEQWFTTQ TEELNKQVVS SSEQLQSYQA EIIELRRTVN ALEIELQAQH NLRDSLENTL TESEARYSSQ LSQVQSLITN
VESQLAEIRS DLERQNQEYQ VLLDVRARLE CEINTYRSLL ESEDCNLPSN PCATTNACSK PIGPCLSNPC TSCVPPAPCT
PCAPRPRCGP CNSFVR

GT3 38.22%

MPYNFCLPSL SCRTSCSSRP CVPPSCHSCT LPGACNIPAN VSNCNWFCEG SFNGSEKETM QFLNDRLASY LEKVRQLERD
NAELENLIRE RSQQQEPLLC PSYQSYFKTI EELQQKILCT KSENARLVVQ IDNAKLAADD FRTKYQTELS LRQLVESDIN
GLRRILDELT LCKSDLEAQV ESLKEELLCL KSNHEQEVNT LRCQLGDRLN VEVDAAPTVD LNRVLNETRS QYEALVETNR
REVEQWFTTQ TEELNKQVVS SSEQLQSYQA EIIELRRTVN ALEIELQAQH NLRDSLENTL TESEARYSSQ LSQVQSLITN
VESQLAEIRS DLERQNQEYQ VLLDVRARLE CEINTYRSLL ESEDCNLPSN PCATTNACSK PIGPCLSNPC TSCVPPAPCT
PCAPRPRCGP CNSFVR

Sequence coverage of KRT81 from each study.

GN=KRT81: Keratin, type II cuticular Hb1 OS=Homo sapiens

T1 76.44%

```
MTCGSGFGGR AFSCISACGP RPGRCCITAA PYRGISCYRG LTGGFGSHSV CGGFRAGSCG RSFGYRSGGV CGPSPPCITT
VSVNESLLTP LNLEIDPNAQ CVKQEEKEQI KSLNSRFAAF IDKVRFLEQQ NKLLETKLQF YQNRECCQSN LEPLFEGYIE
TLRREAECVE ADSGRLASEL NHVQEVLEGY KKKYEEEVSL RATAENEFVA LKKDVDCAYL RKSDLEANVE ALIQEIDFLR
RLYEEEILIL QSHISDTSVV VKLDNSRDLN MDCIIAEIKA QYDDIVTRSR AEAESWYRSK CEEMKATVIR HGETLRRTKE
EINELNRMIQ RLTAEVENAK CQNSKLEAAV AQSEQQGEAA LSDARCKLAE LEGALQKAKQ DMACLIREYQ EVMNSKLGLD
IEIATYRRLL EGEEQRLCEG IGAVNVCVSS SRGGVVCGDL CVSGSRPVTG SVCSAPCNGN VAVSTGLCAP CGQLNTTCGG
GSCGVGSCGI SSLGVGSCGS SCRKC
```

T2 (no KRT81)

T3 75.64%

```
MTCGSGFGGR AFSCISACGP RPGRCCITAA PYRGISCYRG LTGGFGSHSV CGGFRAGSCG RSFGYRSGGV CGPSPPCITT
VSVNESLLTP LNLEIDPNAQ CVKQEEKEQI KSLNSRFAAF IDKVRFLEQQ NKLLETKLQF YQNRECCQSN LEPLFEGYIE
TLRREAECVE ADSGRLASEL NHVQEVLEGY KKKYEEEVSL RATAENEFVA LKKDVDCAYL RKSDLEANVE ALIQEIDFLR
RLYEEEILIL QSHISDTSVV VKLDNSRDLN MDCIIAEIKA QYDDIVTRSR AEAESWYRSK CEEMKATVIR HGETLRRTKE
EINELNRMIQ RLTAEVENAK CQNSKLEAAV AQSEQQGEAA LSDARCKLAE LEGALQKAKQ DMACLIREYQ EVMNSKLGLD
IEIATYRRLL EGEEQRLCEG IGAVNVCVSS SRGGVVCGDL CVSGSRPVTG SVCSAPCNGN VAVSTGLCAP CGQLNTTCGG
GSCGVGSCGI SSLGVGSCGS SCRKC
```

K1 38.81%

```
MTCGSGFGGR AFSCISACGP RPGRCCITAA PYRGISCYRG LTGGFGSHSV CGGFRAGSCG RSFGYRSGGV CGPSPPCITT
VSVNESLLTP LNLEIDPNAQ CVKQEEKEQI KSLNSRFAAF IDKVRFLEQQ NKLLETKLQF YQNRECCQSN LEPLFEGYIE
TLRREAECVE ADSGRLASEL NHVQEVLEGY KKKYEEEVSL RATAENEFVA LKKDVDCAYL RKSDLEANVE ALIQEIDFLR
RLYEEEILIL QSHISDTSVV VKLDNSRDLN MDCIIAEIKA QYDDIVTRSR AEAESWYRSK CEEMKATVIR HGETLRRTKE
EINELNRMIQ RLTAEVENAK CQNSKLEAAV AQSEQQGEAA LSDARCKLAE LEGALQKAKQ DMACLIREYQ EVMNSKLGLD
IEIATYRRLL EGEEQRLCEG IGAVNVCVSS SRGGVVCGDL CVSGSRPVTG SVCSAPCNGN VAVSTGLCAP CGQLNTTCGG
GSCGVGSCGI SSLGVGSCGS SCRKC
```

K2 39.41%

```
MTCGSGFGGR AFSCISACGP RPGRCCITAA PYRGISCYRG LTGGFGSHSV CGGFRAGSCG RSFGYRSGGV CGPSPPCITT
VSVNESLLTP LNLEIDPNAQ CVKQEEKEQI KSLNSRFAAF IDKVRFLEQQ NKLLETKLQF YQNRECCQSN LEPLFEGYIE
TLRREAECVE ADSGRLASEL NHVQEVLEGY KKKYEEEVSL RATAENEFVA LKKDVDCAYL RKSDLEANVE ALIQEIDFLR
RLYEEEILIL QSHISDTSVV VKLDNSRDLN MDCIIAEIKA QYDDIVTRSR AEAESWYRSK CEEMKATVIR HGETLRRTKE
EINELNRMIQ RLTAEVENAK CQNSKLEAAV AQSEQQGEAA LSDARCKLAE LEGALQKAKQ DMACLIREYQ EVMNSKLGLD
IEIATYRRLL EGEEQRLCEG IGAVNVCVSS SRGGVVCGDL CVSGSRPVTG SVCSAPCNGN VAVSTGLCAP CGQLNTTCGG
GSCGVGSCGI SSLGVGSCGS SCRKC
```

K3 61.78%

```
MTCGSGFGGR AFSCISACGP RPGRCCITAA PYRGISCYRG LTGGFGSHSV CGGFRAGSCG RSFGYRSGGV CGPSPPCITT
VSVNESLLTP LNLEIDPNAQ CVKQEEKEQI KSLNSRFAAF IDKVRFLEQQ NKLLETKLQF YQNRECCQSN LEPLFEGYIE
TLRREAECVE ADSGRLASEL NHVQEVLEGY KKKYEEEVSL RATAENEFVA LKKDVDCAYL RKSDLEANVE ALIQEIDFLR
RLYEEEILIL QSHISDTSVV VKLDNSRDLN MDCIIAEIKA QYDDIVTRSR AEAESWYRSK CEEMKATVIR HGETLRRTKE
EINELNRMIQ RLTAEVENAK CQNSKLEAAV AQSEQQGEAA LSDARCKLAE LEGALQKAKQ DMACLIREYQ EVMNSKLGLD
IEIATYRRLL EGEEQRLCEG IGAVNVCVSS SRGGVVCGDL CVSGSRPVTG SVCSAPCNGN VAVSTGLCAP CGQLNTTCGG
GSCGVGSCGI SSLGVGSCGS SCRKC
```

KT1 82.97%

MTCGSGFGGR AFSCISACGP RPGRCCITAA PYRGISCYRG LTGGFGSHSV CGGFRAGSCG RSFGYRSGGV CGPSPPCITT
VSVNESLLTP LNLEIDPNAQ CVKQEEKEQI KSLNSRFAAF IDKVRFLEQQ NKLLETKLQF YQNRECCQSN LEPLFEGYIE
TLRREAECVE ADSGRLASEL NHVQEVLEGY KKKYEEEVSL RATAENEFVA LKKDVDCAYL RKSDLEANVE ALIQEIDFLR
RLYEEEILIL QSHISDTSVV VKLDNSRDLN MDCIIAEIKA QYDDIVTRSR AEAESWYRSK CEEMKATVIR HGETLRRTKE
EINELNRMIQ RLTAEVENAK CQNSKLEAAV AQSEQQGEAA LSDARCKLAE LEGALQKAKQ DMACLIREYQ EVMNSKLGLD
IEIATYRRLL EGEEQRLCEG IGAVNVCVSS SRGGVVCGDL CVSGSRPVTG SVCSAPCNGN VAVSTGLCAP CGQLNTTCGG
GSCGVGSCGI SSLGVGSCGS SCRKC

KT2 86.73%

MTCGSGFGGR AFSCISACGP RPGRCCITAA PYRGISCYRG LTGGFGSHSV CGGFRAGSCG RSFGYRSGGV CGPSPPCITT
VSVNESLLTP LNLEIDPNAQ CVKQEEKEQI KSLNSRFAAF IDKVRFLEQQ NKLLETKLQF YQNRECCQSN LEPLFEGYIE
TLRREAECVE ADSGRLASEL NHVQEVLEGY KKKYEEEVSL RATAENEFVA LKKDVDCAYL RKSDLEANVE ALIQEIDFLR
RLYEEEILIL QSHISDTSVV VKLDNSRDLN MDCIIAEIKA QYDDIVTRSR AEAESWYRSK CEEMKATVIR HGETLRRTKE
EINELNRMIQ RLTAEVENAK CQNSKLEAAV AQSEQQGEAA LSDARCKLAE LEGALQKAKQ DMACLIREYQ EVMNSKLGLD
IEIATYRRLL EGEEQRLCEG IGAVNVCVSS SRGGVVCGDL CVSGSRPVTG SVCSAPCNGN VAVSTGLCAP CGQLNTTCGG
GSCGVGSCGI SSLGVGSCGS SCRKC

KT3 82.57%

MTCGSGFGGR AFSCISACGP RPGRCCITAA PYRGISCYRG LTGGFGSHSV CGGFRAGSCG RSFGYRSGGV CGPSPPCITT
VSVNESLLTP LNLEIDPNAQ CVKQEEKEQI KSLNSRFAAF IDKVRFLEQQ NKLLETKLQF YQNRECCQSN LEPLFEGYIE
TLRREAECVE ADSGRLASEL NHVQEVLEGY KKKYEEEVSL RATAENEFVA LKKDVDCAYL RKSDLEANVE ALIQEIDFLR
RLYEEEILIL QSHISDTSVV VKLDNSRDLN MDCIIAEIKA QYDDIVTRSR AEAESWYRSK CEEMKATVIR HGETLRRTKE
EINELNRMIQ RLTAEVENAK CQNSKLEAAV AQSEQQGEAA LSDARCKLAE LEGALQKAKQ DMACLIREYQ EVMNSKLGLD
IEIATYRRLL EGEEQRLCEG IGAVNVCVSS SRGGVVCGDL CVSGSRPVTG SVCSAPCNGN VAVSTGLCAP CGQLNTTCGG
GSCGVGSCGI SSLGVGSCGS SCRKC

C1 34.85%

MTCGSGFGGR AFSCISACGP RPGRCCITAA PYRGISCYRG LTGGFGSHSV CGGFRAGSCG RSFGYRSGGV CGPSPPCITT
VSVNESLLTP LNLEIDPNAQ CVKQEEKEQI KSLNSRFAAF IDKVRFLEQQ NKLLETKLQF YQNRECCQSN LEPLFEGYIE
TLRREAECVE ADSGRLASEL NHVQEVLEGY KKKYEEEVSL RATAENEFVA LKKDVDCAYL RKSDLEANVE ALIQEIDFLR
RLYEEEILIL QSHISDTSVV VKLDNSRDLN MDCIIAEIKA QYDDIVTRSR AEAESWYRSK CEEMKATVIR HGETLRRTKE
EINELNRMIQ RLTAEVENAK CQNSKLEAAV AQSEQQGEAA LSDARCKLAE LEGALQKAKQ DMACLIREYQ EVMNSKLGLD
IEIATYRRLL EGEEQRLCEG IGAVNVCVSS SRGGVVCGDL CVSGSRPVTG SVCSAPCNGN VAVSTGLCAP CGQLNTTCGG
GSCGVGSCGI SSLGVGSCGS SCRKC

C2 29.31%

MTCGSGFGGR AFSCISACGP RPGRCCITAA PYRGISCYRG LTGGFGSHSV CGGFRAGSCG RSFGYRSGGV CGPSPPCITT
VSVNESLLTP LNLEIDPNAQ CVKQEEKEQI KSLNSRFAAF IDKVRFLEQQ NKLLETKLQF YQNRECCQSN LEPLFEGYIE
TLRREAECVE ADSGRLASEL NHVQEVLEGY KKKYEEEVSL RATAENEFVA LKKDVDCAYL RKSDLEANVE ALIQEIDFLR
RLYEEEILIL QSHISDTSVV VKLDNSRDLN MDCIIAEIKA QYDDIVTRSR AEAESWYRSK CEEMKATVIR HGETLRRTKE
EINELNRMIQ RLTAEVENAK CQNSKLEAAV AQSEQQGEAA LSDARCKLAE LEGALQKAKQ DMACLIREYQ EVMNSKLGLD
IEIATYRRLL EGEEQRLCEG IGAVNVCVSS SRGGVVCGDL CVSGSRPVTG SVCSAPCNGN VAVSTGLCAP CGQLNTTCGG
GSCGVGSCGI SSLGVGSCGS SCRKC

C3 39.21%

MTCGSGFGGR AFSCISACGP RPGRCCITAA PYRGISCYRG LTGGFGSHSV CGGFRAGSCG RSFGYRSGGV CGPSPPCITT
VSVNESLLTP LNLEIDPNAQ CVKQEEKEQI KSLNSRFAAF IDKVRFLEQQ NKLLETKLQF YQNRECCQSN LEPLFEGYIE
TLRREAECVE ADSGRLASEL NHVQEVLEGY KKKYEEEVSL RATAENEFVA LKKDVDCAYL RKSDLEANVE ALIQEIDFLR
RLYEEEILIL QSHISDTSVV VKLDNSRDLN MDCIIAEIKA QYDDIVTRSR AEAESWYRSK CEEMKATVIR HGETLRRTKE
EINELNRMIQ RLTAEVENAK CQNSKLEAAV AQSEQQGEAA LSDARCKLAE LEGALQKAKQ DMACLIREYQ EVMNSKLGLD
IEIATYRRLL EGEEQRLCEG IGAVNVCVSS SRGGVVCGDL CVSGSRPVTG SVCSAPCNGN VAVSTGLCAP CGQLNTTCGG
GSCGVGSCGI SSLGVGSCGS SCRKC

CT1 (no KRT81)

CT2 (no KRT81)

CT3 (no KRT81)

G1 (no KRT81)

G2 49.31%

MTCGSGFGGR AFSCISACGP RPGRCCITAA PYRGISCYRG LTGGFGSHSV CGGFRAGSCG RSFGYRSGGV CGPSPPCITT
VSVNESLLTP LNLEIDPNAQ CVKQEEKEQI KSLNSRFAAF IDKVRFLEQQ NKLLETKLQF YQNRECCQSN LEPLFEGYIE
TLRREAECVE ADSGRLASEL NHVQEVLEGY KKKYEEEVSL RATAENEFVA LKKDVDCAYL RKSDLEANVE ALIQEIDFLR
RLYEEEILIL QSHISDTSVV VKLDNSRDLN MDCIIAEIKA QYDDIVTRSR AEAESWYRSK CEEMKATVIR HGETLRRTKE
EINELNRMIQ RLTAEVENAK CQNSKLEAAV AQSEQQGEAA LSDARCKLAE LEGALQKAKQ DMACLIREYQ EVMNSKLGLD
IEIATYRRLL EGEEQRLCEG IGAVNVCVSS SRGGVVCGDL CVSGSRPVTG SVCSAPCNGN VAVSTGLCAP CGQLNTTCGG
GSCGVGSCGI SSLGVGSCGS SCRKC

G3 42.97%

MTCGSGFGGR AFSCISACGP RPGRCCITAA PYRGISCYRG LTGGFGSHSV CGGFRAGSCG RSFGYRSGGV CGPSPPCITT
VSVNESLLTP LNLEIDPNAQ CVKQEEKEQI KSLNSRFAAF IDKVRFLEQQ NKLLETKLQF YQNRECCQSN LEPLFEGYIE
TLRREAECVE ADSGRLASEL NHVQEVLEGY KKKYEEEVSL RATAENEFVA LKKDVDCAYL RKSDLEANVE ALIQEIDFLR
RLYEEEILIL QSHISDTSVV VKLDNSRDLN MDCIIAEIKA QYDDIVTRSR AEAESWYRSK CEEMKATVIR HGETLRRTKE
EINELNRMIQ RLTAEVENAK CQNSKLEAAV AQSEQQGEAA LSDARCKLAE LEGALQKAKQ DMACLIREYQ EVMNSKLGLD
IEIATYRRLL EGEEQRLCEG IGAVNVCVSS SRGGVVCGDL CVSGSRPVTG SVCSAPCNGN VAVSTGLCAP CGQLNTTCGG
GSCGVGSCGI SSLGVGSCGS SCRKC

GT1 31.29%

MTCGSGFGGR AFSCISACGP RPGRCCITAA PYRGISCYRG LTGGFGSHSV CGGFRAGSCG RSFGYRSGGV CGPSPPCITT
VSVNESLLTP LNLEIDPNAQ CVKQEEKEQI KSLNSRFAAF IDKVRFLEQQ NKLLETKLQF YQNRECCQSN LEPLFEGYIE
TLRREAECVE ADSGRLASEL NHVQEVLEGY KKKYEEEVSL RATAENEFVA LKKDVDCAYL RKSDLEANVE ALIQEIDFLR
RLYEEEILIL QSHISDTSVV VKLDNSRDLN MDCIIAEIKA QYDDIVTRSR AEAESWYRSK CEEMKATVIR HGETLRRTKE
EINELNRMIQ RLTAEVENAK CQNSKLEAAV AQSEQQGEAA LSDARCKLAE LEGALQKAKQ DMACLIREYQ EVMNSKLGLD
IEIATYRRLL EGEEQRLCEG IGAVNVCVSS SRGGVVCGDL CVSGSRPVTG SVCSAPCNGN VAVSTGLCAP CGQLNTTCGG
GSCGVGSCGI SSLGVGSCGS SCRKC

GT2 32.67%

MTCGSGFGGR AFSCISACGP RPGRCCITAA PYRGISCYRG LTGGFGSHSV CGGFRAGSCG RSFGYRSGGV CGPSPPCITT
VSVNESLLTP LNLEIDPNAQ CVKQEEKEQI KSLNSRFAAF IDKVRFLEQQ NKLLETKLQF YQNRECCQSN LEPLFEGYIE
TLRREAECVE ADSGRLASEL NHVQEVLEGY KKKYEEEVSL RATAENEFVA LKKDVDCAYL RKSDLEANVE ALIQEIDFLR
RLYEEEILIL QSHISDTSVV VKLDNSRDLN MDCIIAEIKA QYDDIVTRSR AEAESWYRSK CEEMKATVIR HGETLRRTKE
EINELNRMIQ RLTAEVENAK CQNSKLEAAV AQSEQQGEAA LSDARCKLAE LEGALQKAKQ DMACLIREYQ EVMNSKLGLD
IEIATYRRLL EGEEQRLCEG IGAVNVCVSS SRGGVVCGDL CVSGSRPVTG SVCSAPCNGN VAVSTGLCAP CGQLNTTCGG
GSCGVGSCGI SSLGVGSCGS SCRKC

GT3 35.84%

MTCGSGFGGR AFSCISACGP RPGRCCITAA PYRGISCYRG LTGGFGSHSV CGGFRAGSCG RSFGYRSGGV CGPSPPCITT
VSVNESLLTP LNLEIDPNAQ CVKQEEKEQI KSLNSRFAAF IDKVRFLEQQ NKLLETKLQF YQNRECCQSN LEPLFEGYIE
TLRREAECVE ADSGRLASEL NHVQEVLEGY KKKYEEEVSL RATAENEFVA LKKDVDCAYL RKSDLEANVE ALIQEIDFLR
RLYEEEILIL QSHISDTSVV VKLDNSRDLN MDCIIAEIKA QYDDIVTRSR AEAESWYRSK CEEMKATVIR HGETLRRTKE
EINELNRMIQ RLTAEVENAK CQNSKLEAAV AQSEQQGEAA LSDARCKLAE LEGALQKAKQ DMACLIREYQ EVMNSKLGLD
IEIATYRRLL EGEEQRLCEG IGAVNVCVSS SRGGVVCGDL CVSGSRPVTG SVCSAPCNGN VAVSTGLCAP CGQLNTTCGG
GSCGVGSCGI SSLGVGSCGS SCRKC
